# Mr. Thomas, on Midwifery

**Published:** 1800-11

**Authors:** Sam. Thomas

**Affiliations:** Surgeon. Wakefield, Yorkshire


### *To the Editors of the Medical and Phyſical Journal.*

GENTLEMEN,

IN Number XVIII of your valuable Medical and Phyſical Journal, Dr. Kinglake ſays, that in labours the ſupport of the perinæum is more worthy of female attention than that of an experienced accoucheur, and that the practice of midwifery is not uniformly neceſſary. In anſwer to which, I would ſuppoſe, that Dr. Kinglake has rarely attended to natural labours, or elſe he would certainly never have objected to the ſupport of the perinæum. From the reſpectable authority of Drs. Oſborn and Clark, to whom I am indebted for my ſhare of knowledge in this ſcience, the neceſſity in their lectures was ſtrongly inculcated of making a proper preſſure upon the perinæum, (and ſince then by Dr. Oſborn, in his excellent work upon Natural and Laborious Caſes of Midwifery,) for the benefit of ſecuring it againſt laceration, and at the ſame time that the expulſion of the child will be prevented from haſtily taking place; by which means the placenta will be delivered with more eaſe both to practitioners and patients, and in nineteen times out of twenty will be one means of facilitating its expulſion.

Notwithſtanding what Dr. Kinglake has advanced, the ſupport of the perinæum is probably one of the principal objects in the practice of midwifery, and conſtitutes the greateſt part of a natural labour. The reſiſtance employed by an experienced practitioner, muſt certainly be greater than that perhaps of a kindred female; the benefit greater, and more attention paid to the ſtate of the patient by leſs frequent examinations per vaginam.

The reſiſtance has certainly prevented laceration; and other evils have taken place from a want of a due regard being paid to this ſubject, and have principally happened to ignorant women, denominating themſelves midwifes. If Dr. Kinglake wiſhed midwifery to fall again univerſally into the hands of ignorant women, (which ſeems to be the caſe) this is certainly one way of encouraging it, as by mentioning ſuch circumſtances as theſe, they may militate againſt the general practice.

As the Journal is univerſally read by numbers not of the profeſſion,—from a worthy author of the Qualifications of an Accoucheur, I beg leave to ſelect theſe few remarks: "If inſtances were not known, one would ſcarcely ſuppoſe that any man would be ſo unfeeling and audacious, as to practiſe this branch of the art without a regular training, or that from any compliment compliment hufbands would chufe fuch a perfon to act in this *important office*; the whole affociation of profeffors and practitioners in this department, fhould, for the fake of *humanity*, earneftly unite in difcountenancing untutored intruders; if *guardians of the profession fail to support regularity*, incompetent judges cannot be blamed for making improper diftinctions. Students who are at the expence of a regular education, who clofely apply in acquiring qualifications, and fulfil what is required of them, lofe the neceffary fpur to induftry, when the unqualified can gain equal privileges and equal encouragement.

"Regulars cannot confult with ignorant and untrained pretenders to the art, confiftently with their duty to medical fociety, or with integrity to their employers.

"It may be argued, that women devoid of elementary rules, are frequently trufted with the management of this *momentous office*; but it may be afked, are not men generally preferred, in the expectation of greater fafety in the hands of thofe that have ftudied the ftructure of the parts concerned, and the rules of practice under eminent teachers? Every confcientious profeffor of the art muft acknowledge, that there may be greater hazard from this part of fcience being left to the management of men than women, unlefs fuitable inftructions have been imbibed.

"What a difappointment muft it be to a delicate woman, that has with difficulty being prevailed on to confent to the cuftomary preference, fhould fhe difcover her accoucheur to have no qualifications fuperior to a MIDWIFE."

And again, the worthy Mr. Lucas fays, "As far as many year's experience have enabled me to judge, a regular difciplined accoucheur may find that fome recent information may be derived from every cafe; but fuch proficience cannot be acquired without a proper foundation, a regular education."

Having attended a confiderable number of women, I am of Mr. Lucas's opinion, that fomething may be learned from every cafe; I have attended in midwifery, and I can alfo add, and I think the generality of practitioners will allow, that by a proper management of natural labours, with equal regard to the fupport of the perinæum, &c. they will find that the women recover much better; a good knowledge of natural labour certainly affifts us with information in all cafes of the preternatural; it is frequently to be obferved where one ftage of parturition is unufually rapid, or unnaturally expedited, a fubfequent ftage is retarded; as, for inftance, where the child is fuddenly born from a want of due fupport of perinæum, the placenta is retarded; this I know to be frequently the cafe.

There

There will be great utility in counteracting an hafty procedure, particularly in impatient women, in order that the conclufion of birth may co-operate in affifting the contraction of the uterus, and, confequently, the expulfion of the placenta.

Would not a fenfible, humane practitioner be ferioufly vexed if, by not paying proper attention to the laft ftage of delivery, it fhould be faid by his negligence the woman was injured? Would he have a chance of being employed in the fame family? Would not his compeers, in the fame town, take advantage both of his negligence and his want of fkill?

With regard to the delivery of the placenta, confiderable mifchief has enfued from its immediate expulfion. My practice is, that if the placenta does not come eafily away in the courfe of twenty minutes from the birth of the child, by pulling gently at the funis, in different directions; if, then, there fhould be any retention, I wait another half hour; and, by gently pulling, try if any advances have taken place. If no advances have taken place within an hour or two, (according to circumftances) I then find it neceffary to deliver by manual introduction; and not more than two cafes have taken place in my own practice. But in all cafes we have not the advantage of the funis, by its rupturing from the fmallnefs of its fize. In moft cafes the manual operation is certainly prevented, by paying a proper regard to the fupport of the perinæum, and preventing the hafty expulfion of the fœtus. I am,

Gentlemen,

Your humble fervant,

*Wakefield, Yorkfhire.* SAM. THOMAS, Surgeon.

P. S. Upon mature confideration, I fhould be forry to doubt the abilities of Dr. Kinglake, whofe papers in your ufeful Mifcellany I have read with pleafure, (all except the laft); and, being an advocate for the good fuccefs of the fcience of midwifery, which I think ought to be in the hands of the moft intelligent practitioners, and not in thofe of low uneducated women, it could not efcape my notice without fomething being faid on the fubject, as I think if the perinæum is not properly fupported, laceration may enfue, and perhaps other direful fymptoms take place. In a cafe of the prefentation of the face, or rather the chin, which I had lately, if there had not been due fupport applied, during every pain, to the perinæum, laceration, I am certain, would have taken place.

